# A dihydrofuro[2,3-b] benzofuran derivative alleviates lipopolysaccharide induced acute lung injury *via* suppressing MAPK signaling

**DOI:** 10.3389/fphar.2026.1763318

**Published:** 2026-04-21

**Authors:** Yuxi Wang, Zeping Shan, Shenyan Shao, Yuanyuan Yang, Aijing Zhang, Guimei Wang, Xiaohong Wu, Yuqing Cai, Chengyun Xu

**Affiliations:** 1 Department of Respirology, Hangzhou Lin’an Traditional Chinese Medicine Hospital, Affiliated Hospital, Hangzhou City University, Hangzhou, China; 2 Department of Pharmacology, Hangzhou City University School of Medicine, Hangzhou, China; 3 Department of Respirology, Sir Run Run Shaw Hospital, Zhejiang University School of Medicine, Hangzhou, China; 4 Key laboratory of Quantum Materials Control of Zhejiang Provience, School of Information and Electrical Engineering, Hangzhou City University, Hangzhou, China

**Keywords:** acute lung injury, anti-inflammatory activity, dihydrofurobenzofuran derivatives, MAPK signaling, Rhodomyrtus tomentosa

## Abstract

**Introduction:**

*Rhodomyrtus tomentosa* (Ait.) Hassk. (Myrtaceae) has been traditionally used in Southeast Asia to treat inflammation, fever, and respiratory ailments. However, its bioactive components and molecular mechanisms remain unclear. To design novel dihydrofuro [2,3-b]benzofuran derivatives inspired by *R. tomentosa* constituents and to evaluate their anti-inflammatory activity and mechanism in a lipopolysaccharide (LPS)-induced acute lung injury (ALI) model.

**Methods:**

A series of rhodomentosone-like compounds were screened in LPS-stimulated RAW264.7 macrophages for *IL-1*β and *TNF-*α expression. The most active molecule, QFM-3m, was tested in LPS-induced murine ALI through histopathology, cytokine assays, and vascular permeability analysis. Mechanistic studies were conducted using transcriptomic and protein analyses of primary macrophages.

**Results:**

QFM-3m significantly inhibited LPS-induced *IL-1a*, *IL-1*β*, IL-6* and *TNF-*α expression *in vitro* and attenuated lung inflammation, cytokine production, and vascular leakage *in vivo*. Transcriptomic, western blot and molecular docking analysis revealed that QFM-3m selectively suppressed ERK, JNK, and p38 phosphorylation, while NF-κB activation remained unaffected.

**Conclusion:**

QFM-3m, a novel dihydrofurobenzofuran derivative derived from *R. tomentosa*, exerts potent anti-inflammatory and lung-protective effects through selective inhibition of the MAPK pathway. These findings highlight the therapeutic potential of *R. tomentosa*-inspired scaffolds for inflammatory lung diseases.

## Introduction

Inflammation is a fundamental biological defense mechanism that protects the host from infection and injury; however, uncontrolled or excessive inflammation can lead to tissue damage and organ failure. Acute lung injury (ALI) and its severe form, acute respiratory distress syndrome (ARDS), are life-threatening inflammatory diseases characterized by disruption of the alveolar–capillary barrier, pulmonary edema, and severe hypoxemia (Bos and Ware, 2022; Luh and Chiang, 2007). Despite advances in understanding their pathogenesis, there remains no specific pharmacological therapy that effectively prevents or reverses ALI. Therefore, discovering novel anti-inflammatory agents with potent efficacy and low toxicity remains a pressing need (Mokrá, 2020; Shen et al., 2024).

When exposed to injurious stimuli such as lipopolysaccharide (LPS), innate immune cells-particularly macrophages and neutrophils-are activated and infiltrate the lung parenchyma (Goodman et al., 2003). This infiltration is accompanied by the release of pro-inflammatory cytokines, including tumor necrosis factor-α (TNF-α), interleukin-1α (IL-1α), interleukin-1β (IL-1β), and interleukin-6 (IL-6), which amplify pulmonary inflammation and increase vascular permeability (Aliyu et al., 2022; Finigan, 2009). The resulting uncontrolled inflammatory response leads to progressive tissue damage and impaired gas exchange (Yin et al., 2025).

**FIGURE 1:**
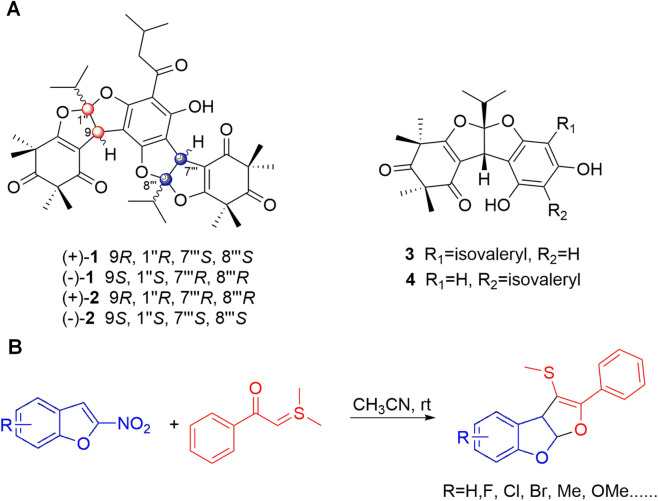
Chemical structures of rhodomentosones and schematic illustration of their structural modification. **(A)** Chemical structures of (dihydro)furo-fused heterocycles derivatives 1–4 isolated from plant. **(B)** Our previous work focused on dihydrofuro [2,3-b]benzofuran based small molecules.

Medicinal plants have historically served as a valuable source of pharmacologically active natural products, many of which have inspired modern drug discovery (Newman and Cragg, 2020). *Rhodomyrtus tomentosa* (Ait.) Hassk. (Myrtaceae), commonly known as “Rose Myrtle,” is a traditional medicinal plant widely distributed in Southeast Asia. In folk medicine, its leaves and fruits have been used to treat fever, diarrhea, wounds, and inflammatory disorders (Vo-An et al., 2025; Nguyen et al., 2004). Phytochemical studies have revealed that *R. tomentosa* is rich in polyphenols, triterpenoids, and benzofuran-type meroterpenoids, which exhibit diverse bioactivities, including antibacterial, antioxidant, and anti-inflammatory effects (Zhou et al., 2018; Wang et al., 2022). Notably, meroterpenoids such as rhodomyrtone and rhodomentosones have shown inhibitory effects on inflammatory pathways, including MAPK and NF-κB signaling in activated macrophages (Tayeh et al., 2017; Jeong et al., 2013; Chorachoo et al., 2018). Deng et al. identified a series of novel (dihydro)furo-fused heterocycles derivatives, rhodomentosones A (Bos and Ware, 2022) and B (Luh and Chiang, 2007), along with their biogenetically related dimers (Mokrá, 2020; Shen et al., 2024) (Figure 1A) (Deng et al., 2021). The (dihydro)furo-fused heterocycles motif represents an important privileged scaffolds frequently encountered in biological natural products and synthetic drug candidates (Zhang et al., 2020; Fang et al., 2023; Zhang et al., 2021). Inspired by these findings, our group designed and synthesized several dihydrofuro [2,3-b]benzofuran based small molecules that mimic the core architecture of rhodomentosones (Figure 1B) (Qiu et al., 2023). This study screened these dihydrofuro [2,3-b]benzofuran structurally modified compounds and explored their protective effects and mechanisms against acute lung injury in mice.

## Materials and methods

### Cell cultures and treatments

The RAW264.7 murine macrophage cell line was obtained from the American Type Culture Collection (ATCC, Manassas, VA, United States) and maintained in RPMI-1640 medium supplemented with 10% fetal bovine serum (FBS), 100 μg/mL streptomycin, and 100 U/mL penicillin (Life Technologies, Carlsbad, CA, United States). Cells were seeded into 24-well plates and allowed to adhere for 12 h. Upon reaching approximately 70% confluence, cells were serum-starved prior to treatment. For compound screening, cells were pretreated with individual compounds (10 μM), kindly provided by Dr. Jiankang Zhang (Hangzhou City University), for 1 h, followed by stimulation with LPS (100 ng/mL; Sigma-Aldrich, United States). To evaluate the anti-inflammatory effects of QFM-3m, cells were pretreated with QFM-3m at concentrations of 2.0 or 5.0 μM for 1 h prior to LPS stimulation. After 12 h of LPS exposure, total RNA was extracted for subsequent gene expression analysis.

### Cell viability measurement by CCK-8 assay

The RAW264.7 murine macrophage cells were seeded in the 96-well plate to adhere 12 h. When cell confluence reached approximately 70%, they were serum-starved. Subsequently, the cells were pretreated with QFM-3m at concentrations of 0, 0.1, 0.25, 1.0, 2.5, 5.0 or 10.0 μM for 72 h, respectively. The Cell Counting Kit-8 (CCK-8) solution was added to each well of the plate according to the CCK-8 kit (Biosharp, Guangzhou, China). Then the plate was measured at 450 nm using a spectrophotometer (TECAN, Switzerland).

### Mice handling and LPS-induced mice lung injury model

Eight-week-old male C57BL/6J mice (body weight: 20 ± 2.0 g) were purchased from Qizhen Laboratory Animal Center (Hangzhou, China). A total of 40 mice were randomly assigned into five groups (n = 8 per group): (Bos and Ware, 2022): Control group, (Luh and Chiang, 2007), LPS group, (Mokrá, 2020), LPS + QFM-3m (0.3 mg/mL) group, (Shen et al., 2024), LPS + QFM-3m (0.9 mg/mL) group, and (Goodman et al., 2003) LPS + dexamethasone group. The LPS-induced ALI model was established following a standard protocol (Hussain et al., 2019). Adult C57BL/6J mice were anesthetized with isoflurane (induction at 4%, 1%–2% for maintenanceand delivered at a flow rate of 0.8 L/min), and under fiber-optic illumination, an atomizing needle (Huironghe, Beijing, China) was inserted into the airway to administer nebulized LPS (4 mg/mL, 10 μL per 10 g body weight). Mice in the control group received an equivalent volume of sterile saline. QFM-3m was administered at concentrations of 0.3 or 0.9 mg/mL (10 μL per 10 g body weight) using the same procedure 12 h before and 2 h after LPS challenge. Dexamethasone (1 mg/kg) was administered intraperitoneally 2 h after LPS instillation. Mice were euthanized 24 h after modeling by gradual exposure to carbon dioxide (CO_2_) at a displacement rate of 30% of the chamber volume per minute. After respiratory arrest was observed, CO_2_ exposure was maintained for an additional 2–3 min, and death was subsequently confirmed by cervical dislocation. Bronchoalveolar lavage fluid (BALF) and lung tissues were collected for subsequent analyses.All animals were housed at the Hangzhou City University Animal Care Facility according to the Institutional Guidelines for Laboratory Animals, and the animal protocol was approved by the Hangzhou City University Institutional Animal Care and Use Committee (HZCU25083).

### Preparation of BALFs

The bronchoalveolar lavage fluid (BALF) of anesthetized mice were obtained *via* tracheal tube and washing the lung with 1.0 mL of sterilized normal saline containing 500 IU/L heparin (Sigma) three times. BALFs were diluted with 1.0 mL saline containing 2% fetal calf serum (Life Technologies, Carlsbad, CA), and were centrifuged at 500 × *g* at 4 °C for 10 min. The cell pellets were resuspended with saline for Wright Giemsa staining, cell counting and classification.

### Inflammatory cytokines assessment *via* ELISA

Expression levels of inflammatory cytokines (IL-1α, IL-1β, TNF-α, IL-6 (Lianke bio.LTD., Hangzhou, China) in the BALF were assessed by respective ELISA determination kits according to the manufacturer’s instructions. After measurements of the optical density at 450 nm (TECAN, Switzerland), expression of contents was calculated *via* standard curves.

### Lung histopathology

Lung tissue sectioning and staining were performed as previously described (Yao et al., 2011). The inferior lobe of the left lung was fixed in 10% neutral-buffered formalin for 3 days, dehydrated, and embedded in paraffin. The paraffin blocks were trimmed to an appropriate depth to optimize the exposure of airways and alveoli, and 4-μm-thick sections were prepared to stained with Hematoxylin-eosin (H&E). After H&E staining, inflammatory cell infiltration and pathological damage were examined and semi-quantitatively scored.

### NF-κB luciferase reporter assay

HEK293T cells stably transfected with an NF-κB firefly luciferase reporter (kindly provided by Prof. Ximei Wu, Zhejiang University) were seeded into 24-well plates and allowed to adhere for 12 h. Upon reaching approximately 70% confluence, cells were serum-starved prior to treatment. Cells were then pretreated with QFM-3m at concentrations of 2.5 or 5.0 μM for 1 h, followed by stimulation with TNF-α (10 ng/mL; Sigma-Aldrich, United States) for 5 h. Cell lysates were prepared using 100 μL of reporter lysis buffer (Promega, Madison, WI, United States), and 20 μL of the supernatant was used to measure luciferase activity according to the manufacturer’s instructions.

### Western blot

Total protein was extracted from RAW264.7 cells or lung tissues using whole cell lysis buffer supplemented with a protease and phosphatase inhibitor mixture. Protein concentrations were determined, and equal amounts of protein were separated by SDS-PAGE and subsequently transferred onto nitrocellulose (NC) membranes (Millipore, Bedford, MA, United States). Membranes were blocked with 5% (w/v) BSA in PBS for 1 h at room temperature and then incubated overnight at 4 °C with primary antibodies diluted according to the manufacturer’s recommendations: p-p38, p38, p-ERK, ERK, p-JNK, JNK, p-p65, p65 (Huaan-bio, Hangzhou, China), and β-actin (Santa Cruz Biotechnology, Santa Cruz, CA, United States). After washing three to four times with TBST (5 min each), membranes were incubated with HRP-conjugated secondary antibodies (Huaan-bio, Hangzhou, China) for 2 h at room temperature. The protein bands were visualized using an enhanced chemiluminescence (ECL) detection system (Thermo Fisher Scientific) and imaged using a chemiluminescence imaging system (Taton, China). Semi-quantitative analysis of band intensities was performed using ImageJ software (NIH, http://rsb.info.nih.gov/ij/). The intensity of the control group was normalized to 1 for comparison.

### Determination of MPO activity

The myeloperoxidase (MPO) test kit (Nanjing Jiancheng Bioengineering Institute, Nanjing, China) was used to measure MPO activity in lung tissue. Briefly, lung tissue was accurately weighted and homogenized with prepared homogenate medium. Then the supernatant was then taken for MPO activity detection and BCA assay was used to determine the total protein content in the sample.

### *In vivo* pulmonary vascular permeability assessment

Pulmonary microvascular permeability was evaluated by measuring Evans blue dye extravasation, as previously described (Wu et al., 2024). Briefly, mice subjected to LPS-induced acute lung injury were intravenously injected with Evans blue dye (50 mg/kg) *via* the tail vein 1 h prior to euthanasia. Subsequently, the pulmonary vasculature was flushed with normal saline through the right ventricle to remove intravascular dye. The right lung was carefully excised and incubated in formamide solution at room temperature. After 24 h, the samples were centrifuged at 500 × *g* for 10 min at 4 °C. The absorbance of Evans blue extracted from the supernatant was measured at 620 nm using a spectrophotometer (TECAN, Switzerland), and the results were expressed as micrograms of dye per 100 mg of wet lung tissue.

### Primary macrophage isolation

Primary peritoneal macrophages were isolated from 8-week-old C57BL/6J mice as previously described (Wu et al., 2024). Briefly, mice were injected intraperitoneally with 1 mL of 3% thioglycollate (Thermo, United States) for 3 days. The mice were euthanized and their peritoneal cavity was lavaged with 5 mL of ice-cold sterile PBS to collect resident macrophages, respectively. Cells were pelleted by centrifugation at 500 × for 10 min at 4 °C, resuspended in complete RPMI-1640 medium for 2 h. Non-adherent cells were removed and adherent macrophages were used for subsequent experiments. Primary macrophages were seeded into 6-well or 12-well plates and serum-starved overnight prior to treatment. For RNA sequencing analysis, cells were pretreated with QFM-3m (5 μM) for 1 h, followed by stimulation with LPS (100 ng/mL) for 12 h. Cells were then harvested, and total RNA was extracted for transcriptomic analysis. For signaling pathway validation, cells were pretreated with QFM-3m at concentrations of 2.5 or 5.0 μM for 1 h prior to LPS stimulation (100 ng/mL). After 30 min of LPS exposure, cells were collected for total protein extraction and subsequent analysis.

### Quantitative RT-PCR

The cell pellets were collected and lysed by TRIzol reagent (Vazyme Biotechnology Co., Ltd., Hangzhou, China). Total RNA was isolated following the manufacturer’s instruction. Total RNA was reversely transcribed by using SuperScript III reagent (Vazyme Biotechnology Co., Ltd., Hangzhou, China). The mRNA of interest genes was normalized to β-actin, and differences in mRNA were calculated by the 2^−ΔΔCt^. The primers, produced in Shanghai Bioengineering Ltd., Shanghai, China are shown in Supplementary Table S1.

### RNA sequence

RNA sequence was performed by LC-Bio Technology (Hangzhou, China). Briefly, primary macrophages were treated with LPS and/or QFM-3m for 24 h, after which total RNA was extracted and purified using TRIzol reagent (Invitrogen) according to the manufacturer’s instructions. Libraries were sequenced *via* 2 × 150 bp paired-end sequencing on the Illumina NovaSeq 6000 (LC-Bio Technology) following the vendor’s recommended protocol. Differential expression analysis (DEG) and pathway enrichment were performed using standard pipelines provided by LC-Bio (e.g., DESeq2/edgeR and KEGG enrichment).

### Molecular docking analysis

To evaluate the potential interactions between QFM-3m and MAPK kinases, molecular docking was performed using the Schrödinger Maestro suite (Schrödinger, LLC, United States). The crystal structures of p38 MAPK (PDB ID: 1KV2), ERK2 (PDB ID: 5K4I), and JNK (PDB ID: 8ENJ) were obtained from the Protein Data Bank. Proteins were prepared using the Protein Preparation Wizard by removing water molecules, adding hydrogens, and performing energy minimization. The ligand QFM-3m was prepared using LigPrep under the OPLS4 force field. Docking grids were defined based on the active sites of the co-crystallized ligands, and molecular docking was carried out using the Glide module in standard precision (SP) mode. The best docking poses were selected based on GlideScore, and ligand–protein interactions were analyzed accordingly.

### Statistical analysis

All the numerical data of this experiment are expressed as Mean ± standard deviation (SD). The SPSS software (version 22.0, SPSS Inc., Chicago, Ill) is used for statistical analysis. Data were tested for normality (Shapiro-Wilk test) and homogeneity of variance (Levene’s test) prior to analysis. For comparisons between two groups, an unpaired two-tailed Student’s t-test was used. For multiple group comparisons, one-way ANOVA was performed followed by Dunnett’s test. The number of independent biological replicates (n) is indicated in the Methods and figure legends. A value of *p* < 0.05 was considered statistically significant.

## Results

### Screening of potential anti-inflammatory compounds

**FIGURE 2:**
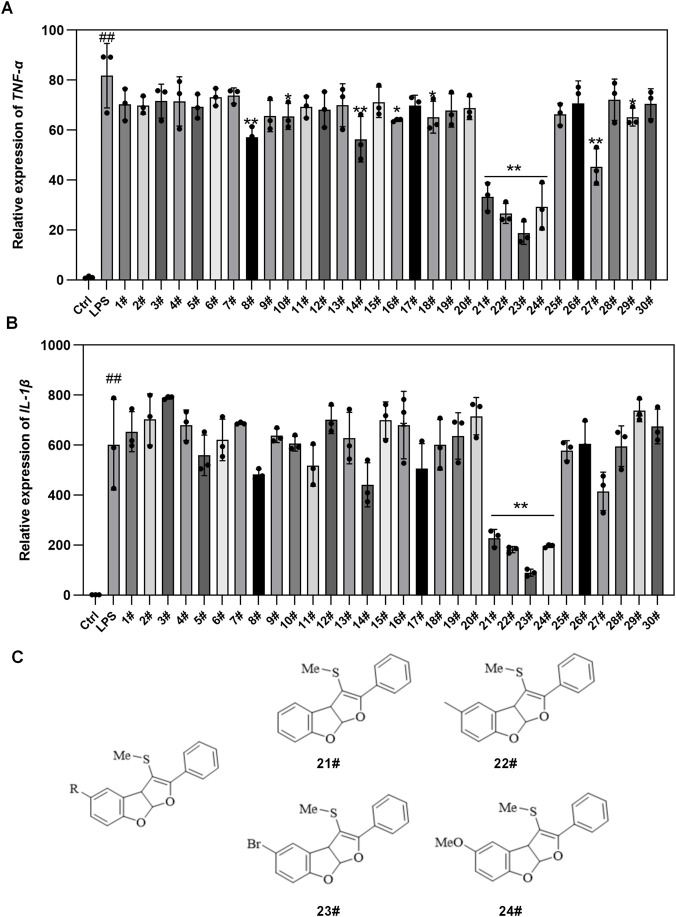
Screening of Potential Anti-inflammatory Compounds. RAW264.7 cells were pretreated with potential anti-inflammatory compounds No. 1-No. 30 at concentrations of 10.0 μM for 1 h, followed by stimulation with LPS (100 ng/mL). After 12 h of LPS stimulation, total RNA was extracted for quantitative RT-PCR of *IL-1*β **(A)** and *TNF-*α **(B)**. The core structures of compounds 21–24 and their corresponding structures **(C)**. n = 3, ^##^ *p* < 0.01 *versus* control Group, ^*^ *p* < 0.05, ^**^ *p* < 0.01 *versus* LPS group.

Compounds from an in-house chemical library were evaluated for anti-inflammatory activity in serum-starved RAW264.7 macrophages. The mRNA expression levels of *IL-1*β and *TNF-*α were quantified by quantitative RT-PCR. Among the tested candidates, compounds 21, 22, 23, and 24 significantly suppressed LPS-induced *IL-1*β and *TNF-*α expression, each showing inhibition rates exceeding 50%, indicative of pronounced anti-inflammatory potential (Figures 2A,B). Structural analysis revealed that these active molecules share a dihydrofuro [3,2-b]benzofuran core, differing only in the substituent at the 5-position of the benzene ring-hydrogen, methyl, bromine, or methoxy (Figure 2C). Notably, compound 23, bearing a bromine substituent (designated QFM-3m), exhibited the most potent inhibitory effect on pro-inflammatory cytokine expression and was therefore selected as the lead compound for subsequent mechanistic and *in vivo* studies.

### QFM-3m ameliorates LPS-induced inflammatory cytokine expression in RAW264.7 cells

**FIGURE 3:**
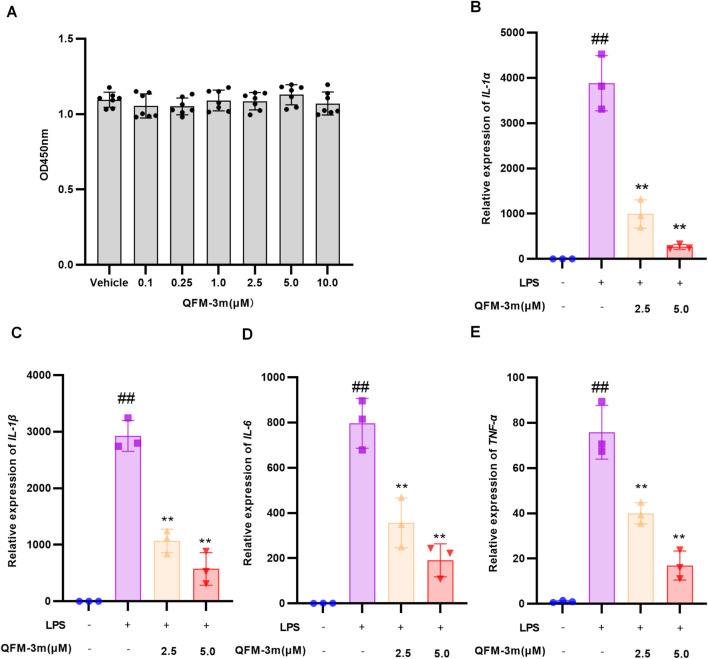
Effect of QFM-3m on LPS induced inflammatory cytokine expression in RAW264.7 cells. **(A)** After RAW264.7 cells were treated with various concentrations of QFM-3m for 72 h, the cell viability was analyzed *via* CCK8 assay. RAW264.7 cells were pretreated with QFM-3m at concentrations of 2.5 μM or 5.0 μM for 1 h, followed by stimulation with LPS. After 12 h of LPS stimulation, total RNA was extracted for quantitative RT-PCR of *IL-1*α **(B)**, *IL-1*β **(C)**, *IL-6* **(D)** and *TNF-*α **(E)**. n = 3, ^##^ *p* < 0.01 *versus* control Group, ^*^ *p* < 0.05, ^**^ *p* < 0.01 *versus* LPS group.

The effect of QFM-3m on the viability of RAW264.7 cells was measured using the CCK8 assay. The results showed that treatment with 0–10 µM QFM-3m did not have any impact on the viability of RAW264.7 cells (Figure 3A). We further evaluated the effect of QFM-3m on LPS-induced inflammatory cytokine expression using quantitative RT-PCR. The results showed that QFM-3m inhibited LPS-induced *IL-1*α, *IL-1*β, *TNF-*α, and *IL-6* expression in a dose-dependent manner at 2.5 and 5.0 μM, with inhibition rates exceeding 80% for all four cytokines at the 5.0 μM dose (Figures 3B–E).

### QFM-3m ameliorates LPS-induced acute lung injury in mice

**FIGURE 4:**
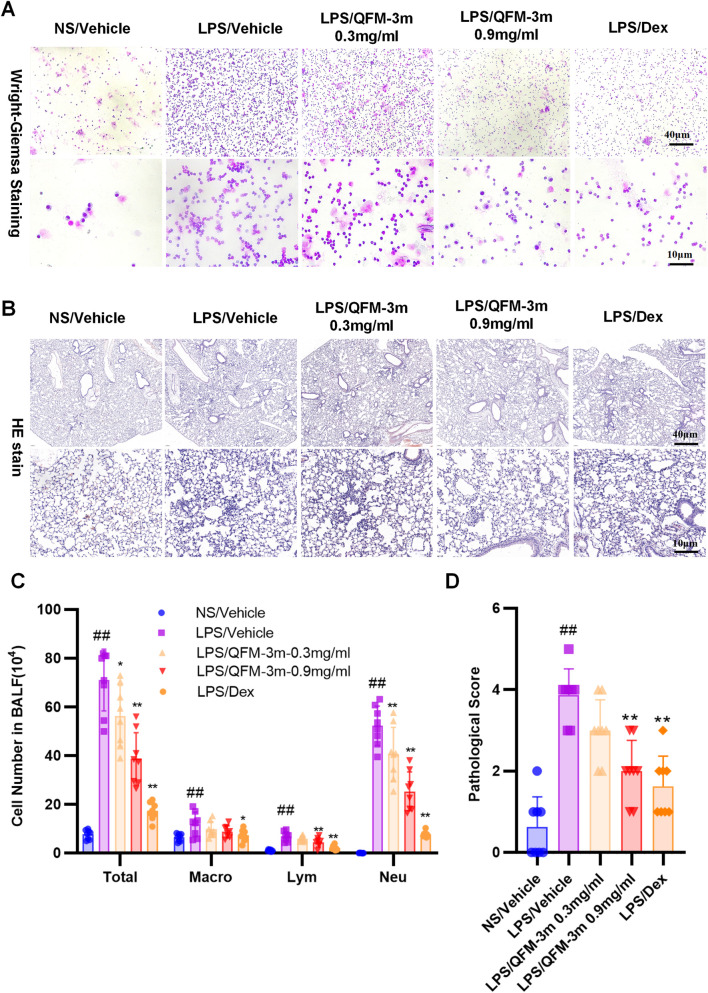
Effect of QFM-3m on BALF inflammatory cell counting, classification and histopathological changes in the LPS-induced ALI mice. Mice were challenged with LPS and treated with QFM-3m (0.3 or 0.9 mg/mL) or dexamethasone. **(A,C)** Total cell counts and differential cell analysis in BALF by Wright–Giemsa staining. **(B,D)** Histopathological changes in lung tissues assessed by H&E staining and histopathological injury score. n = 8, ^##^ *p* < 0.01 *versus* NS/Vehicle group, ^*^ *p* < 0.05, ^**^ *p* < 0.01 *versus* LPS/Vehicle group.

BALF was collected 24 h after LPS airway nebulization to assess inflammatory cell counts and differential cell populations. Compared with the control group, LPS nebulization significantly increased the numbers of macrophages, neutrophils, and lymphocytes in BALF, with neutrophils accounting for 83% of the total inflammatory cells (Figures 4A,C). Treatment with the positive control dexamethasone (1 mg/kg) markedly inhibited LPS-induced inflammatory cell infiltration in BALF. Airway nebulization of QFM-3m at 0.3 and 0.9 mg/mL dose-dependently reduced infiltration of all inflammatory cell types, with significant suppression of total BALF cells and neutrophils. QFM-3m treatment resulted in a reduction of total cell counts by 23.7% and 50.9% at concentrations of 0.3 and 0.9 mg/mL, respectively, while neutrophil counts were decreased by 22.23% and 51.5%, respectively (Figures 4A,C). Histological examination of lung tissue with H&E staining showed that LPS instillation induced extensive inflammatory cell accumulation within alveoli, accompanied by alveolar wall thickening and disruption. Both intraperitoneal dexamethasone and airway nebulization of QFM-3m at 0.3 and 0.9 mg/mL significantly alleviated LPS-induced lung tissue damage (Figures 4B,D).

### QFM-3m attenuates LPS-indued cytokine scretion, vascular hyperpermeability and MPO activity

**FIGURE 5:**
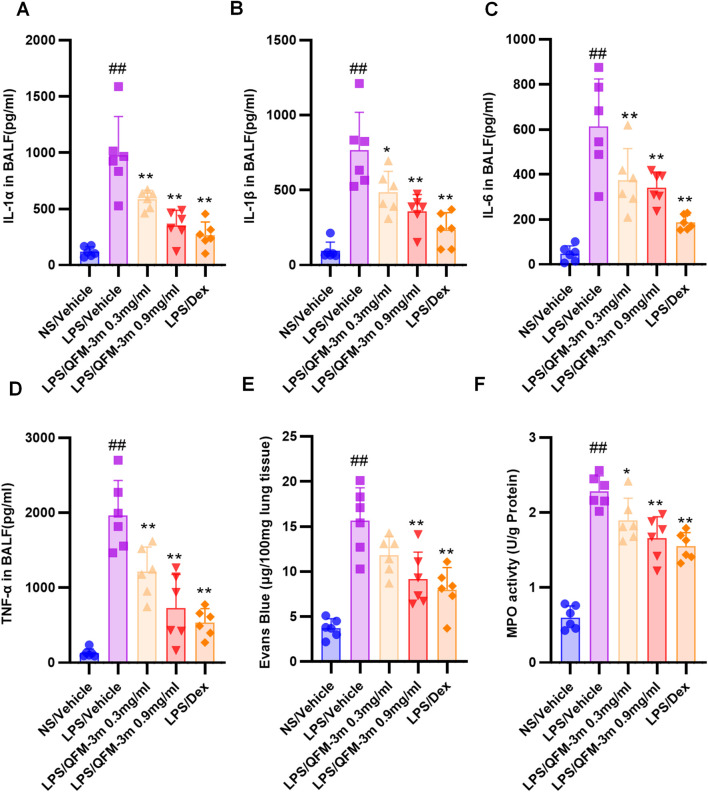
Effect of QFM-3m on LPS-indued cytokine scretion, vascular hyperpermeability and MPO activity. BALF was collected and analysed for IL-1α **(A)**, IL-1β **(B)**, IL-6 **(C)** and TNF-α **(D)** using their respective ELISA kits. **(E)** Evans blue dye (50 mg/kg) was injected into the caudal vein of all mice 1 h before euthanasia. Pulmonary vascular permeability was assessed by determining the accumulation of Evans blue dye in the lung tissue. **(F)** MPO activity from lung homogenates was measured using MPO kits. n = 6, ^##^ *p* < 0.01 *versus* NS/Vehicle group, ^*^ *p* < 0.05, ^**^ *p* < 0.01 *versus* LPS/Vehicle group.

LPS-induced ALI is characterized by excessive production of inflammatory cytokines. To assess this response, the concentrations of IL-1α, IL-1β, TNF-α, and IL-6 in BALF were quantified using ELISA kits. Compared with the control group, LPS exposure markedly elevated the levels of these cytokines. Treatment with QFM-3m at 0.3 and 0.9 mg/mL, as well as dexamethasone (1 mg/kg), significantly reduced cytokine secretion in a dose-dependent manner (Figures 5A–D). These results suggest that QFM-3m confers protection against LPS-induced ALI by alleviating inflammatory responses mediated by proinflammatory cytokines and neutrophil-attracting chemokines. Because vascular endothelial barrier dysfunction is a hallmark of ALI, pulmonary microvascular permeability was further examined using the Evans blue extravasation assay. The amount of Evans blue in BALF was markedly increased after LPS administration compared with the control group, whereas treatment with QFM-3m (0.3 and 0.9 mg/mL) or dexamethasone (1 mg/kg) significantly reduced Evans blue leakage (Figure 5E). These findings indicate that QFM-3m mitigates the LPS-induced increase in pulmonary microvascular permeability. MPO, a key enzyme released by activated neutrophils, serves as a biochemical indicator of neutrophil infiltration and tissue injury. Consistent with inflammatory activation, MPO activity in lung tissue was significantly elevated in LPS-treated mice relative to controls. In contrast, administration of QFM-3m (0.3 and 0.9 mg/mL) or dexamethasone (1 mg/kg) markedly suppressed the increase in MPO activity in a dose-dependent fashion (Figure 5F). Together, these results demonstrate that QFM-3m effectively limits neutrophil recruitment to the alveolar and interstitial compartments, thereby reducing lung inflammation and tissue damage.

### QFM-3m suppresses inflammatory cytokine expression by inhibiting the MAPK signaling pathway

**FIGURE 6:**
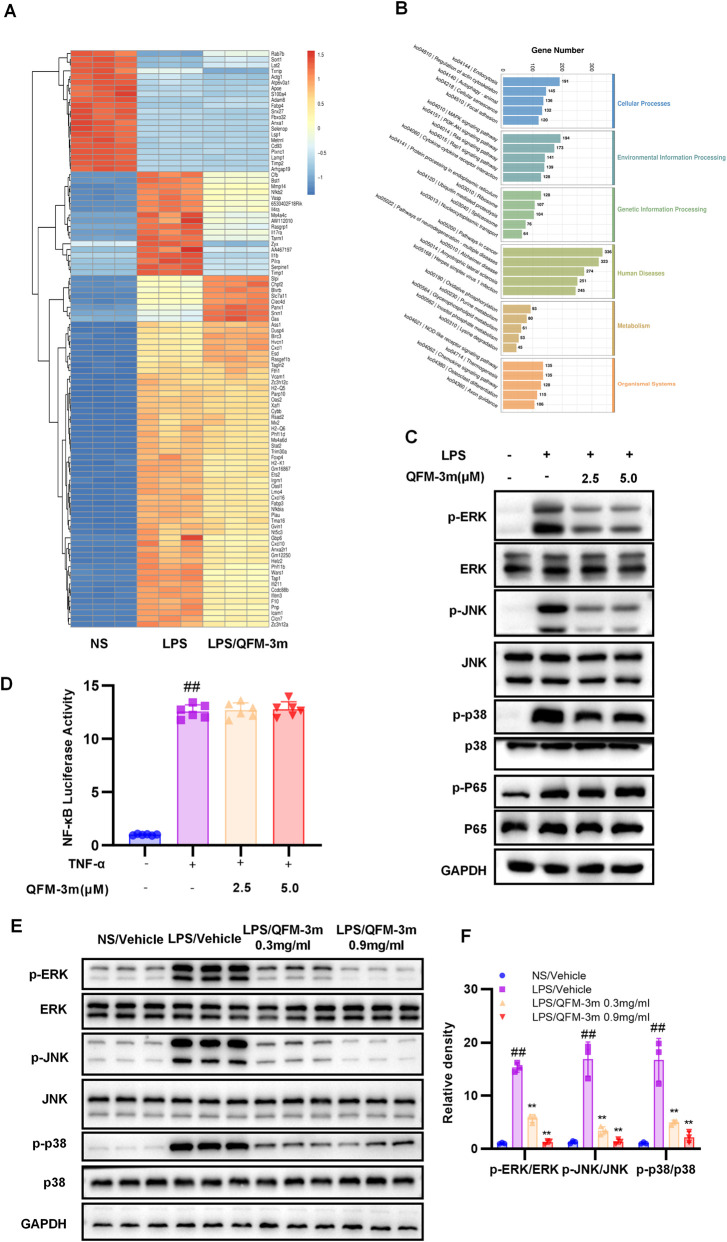
Effect of QFM-3m on the activation of MAPK signaling pathway in response to LPS-stimulation in primary macrophages or lungs. Primary macrophages were stimulated with LPS or LPS plus QFM-3m at 5.0 μM and total RNA was extracted for RNA sequencing. **(A)** Heatmap of the 100 most significantly altered genes among Vehicle, LPS and LPS/QFM-3m. **(B)** KEGG pathway enrichment analysis of the differentially expressed genes. **(C)** Primary macrophages were pretreated with QFM-3m and followed by stimulation with LPS. Total protein was extracted for Western blot detection of phosphorylation level of ERK, JNK, p38 and p65. **(D)** HEK293T cells stably expressing an NF-κB firefly luciferase reporter were pretreated with QFM-3m and stimulated with TNF-α, followed by measurement of luciferase activity. **(E,F)** Preserved lung tissues were extracted the total protein and subjected to Western blot detection of phosphorylation level of ERK, JNK, p38. n = 3 or 6, ^##^ *p* < 0.01 *versus* NS/Vehicle group, ^**^ *p* < 0.01 *versus* LPS/Vehicle group.

To investigate the mechanism underlying the anti-inflammatory effects of QFM-3m, RNA sequencing was performed on primary macrophages stimulated with LPS or LPS plus QFM-3m. DEGs were identified, and the significantly altered genes between LPS and LPS/QFM-3m treatments were selected for further analysis (Figure 6A; Supplementary Table S2). Kyoto Encyclopedia of Genes and Genomes (KEGG) pathway enrichment analysis was conducted to identify major signaling pathways associated with these DEGs. A scatter plot illustrated the top 10 enriched KEGG pathways. Among the signaling modules, the top five pathways were MAPK signaling, PI3K-Akt signaling, Ras signaling, Rap1 signaling, and cytokine-cytokine receptor interaction, with the MAPK pathway containing the largest number of DEGs (194 genes) (Figure 6B). We further analyzed protein levels in primary macrophages by Western blot. The results showed that QFM-3m dose-dependently inhibited LPS-induced phosphorylation of the key MAPK proteins ERK, JNK, and p38. In contrast, the phosphorylation level of P65, a critical mediator of the NF-κB signaling pathway involved in LPS-induced inflammatory cytokine production, was unaffected by QFM-3m treatment (Figure 6C). Consistently, QFM-3m did not significantly alter TNF-α-induced NF-κB luciferase activity in HEK293T cells (Figure 6D). We also examined the activation status of the MAPK signaling pathway in lung tissues from mice with LPS-induced acute lung injury following QFM-3m treatment. The results showed that airway nebulization of QFM-3m at 0.3 and 0.9 mg/mL dose-dependently suppressed LPS-induced phosphorylation of ERK, JNK, and p38 in lung tissue (Figures 6E,F).

### Molecular docking analysis suggests potential interaction of QFM-3m with MAPK kinases

**FIGURE 7:**
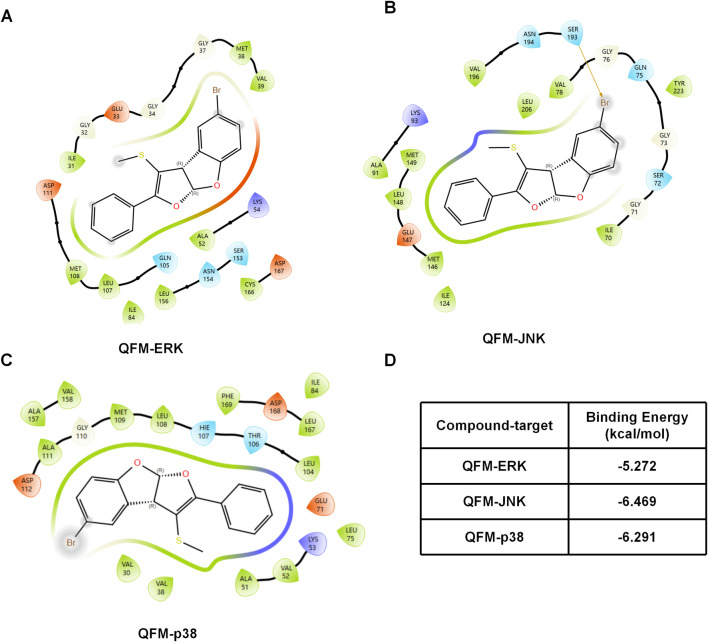
Molecular docking analysis of QFM-3m with MAPK kinases. **(A–C)** Predicted binding modes of QFM-3m within the active pockets of ERK, JNK and p38 MAPK, respectively. QFM-3m exhibited favorable spatial complementarity with the catalytic sites of these kinases. **(D)** GlideScore values of QFM-3m docked to p38, ERK2, and JNK, indicating moderate binding affinity.

To further investigate the potential interactions between the small-molecule compound QFM-3m and key kinases in the MAPK signaling pathway, including p38, ERK1/2, and JNK, molecular docking simulations were performed. The docking results demonstrated that QFM-3m exhibited favorable spatial complementarity with the active pockets of p38, ERK2, and JNK (Figures 7A–C). GlideScore evaluation showed that QFM-3m had a docking score of −5.272∼−6.469 kcal/mol (Figure 7D), suggesting a moderate binding affinity toward these MAPK kinases. These findings indicate that QFM-3m may potentially interact with multiple components of the MAPK signaling pathway, providing structural support for the experimentally observed modulation of MAPK signaling.

## Discussion

The traditional use of *R. tomentosa* for inflammatory conditions provided the ethnopharmacological rationale for this study. Previous phytochemical work has revealed that the leaves contain benzofuran-type meroterpenoids with anti-inflammatory and antimicrobial activity. Herein, we identified a novel class of dihydrofuro [2,3-b] benzofuran-based compounds with robust anti-inflammatory activity. Among these derivatives, QFM-3m bearing a 5-bromo substituent on the benzene ring emerged as the lead compound. Our findings show that QFM-3m attenuates pulmonary infiltration of inflammatory cells, reduces pro-inflammatory cytokine secretion, decreases vascular permeability, and alleviates histopathological damage in a dose-dependent manner. Mechanistically, QFM-3m suppresses the phosphorylation of ERK, JNK, and p38, indicating that its protective effect is closely associated with inhibition of MAPK signaling pathway activation.

Inflammation-driven injury is a hallmark of ALI pathogenesis (Goodman et al., 2003; Kumar, 2020). Previous studies have established that the overproduction of cytokines such as TNF-α and IL-1β, along with excessive recruitment of macrophages and neutrophils, contributes to the disruption of the alveolar-capillary barrier and subsequent pulmonary edema (Zhu et al., 2025). Our results are consistent with this paradigm, as LPS stimulation induced robust cytokine release and inflammatory cell infiltration, both of which were effectively suppressed by QFM-3m. These observations highlight the therapeutic relevance of targeting inflammatory mediators in ALI.

LPS is commonly employed to induce ALI in experimental models because it activates multiple proinflammatory signaling cascades (Zheng et al., 2024). Among these, the MAPK, NF-κB, and phosphoinositide 3-kinase (PI3K)/Akt pathways have been recognized as central regulators of inflammatory responses and tissue injury (Aliyu et al., 2022). Activation of MAPK family members, including ERK1/2, JNK, and p38, promotes the phosphorylation of transcription factors such as AP-1, thereby enhancing the transcription of inflammatory cytokines and chemokines (Shi et al., 2017). NF-κB signaling also plays a critical role in the innate immune response, facilitating the nuclear translocation of NF-κB dimers and subsequent transcription of genes encoding TNF-α, IL-1β, and IL-6 (Izadparast et al., 2022). In parallel, the PI3K/Akt pathway contributes to the regulation of cellular survival and inflammation, forming a complex crosstalk network with MAPK and NF-κB signaling during ALI progression (Ma and Nicolet, 2023).

In the present study, the transcriptomic and cellular experiments consistently demonstrated that QFM-3m exerted a pronounced inhibitory effect on all three major MAPK components-ERK1/2, JNK, and p38-under LPS-induced conditions. This suppression correlated with the observed reduction in proinflammatory cytokine secretion, decreased pulmonary microvascular permeability, and diminished neutrophil infiltration. Notably, QFM-3m did not significantly alter NF-κB activation, as reflected by unchanged levels of phosphorylated p65 and NF-κB luciferase activity, suggesting that its anti-inflammatory effects are primarily mediated through selective MAPK modulation. One possible explanation is that MAPK signaling may play a dominant regulatory role in the early amplification of inflammatory responses under the experimental conditions used in this study, while NF-κB activation may remain relatively stable or be regulated through alternative upstream mechanisms.

To further explore the molecular basis underlying this selective regulation, molecular docking analysis was performed to evaluate the potential interaction between QFM-3m and key MAPK kinases. The docking results indicated that QFM-3m exhibited favorable spatial complementarity with the catalytic pockets of ERK1/2, JNK, and p38, with a GlideScore around −6.0 kcal/mol, suggesting a moderate binding affinity toward these kinases. These findings provide structural support for the experimental observations and suggest that QFM-3m may interact with multiple MAPK signaling components, thereby contributing to the coordinated suppression of MAPK pathway activation. From a structural perspective, the bromine substitution at the 5-position of the benzene ring in QFM-3m may play an important role in its biological activity. Halogen substitution is known to influence molecular polarity, hydrophobicity, and steric interactions, which can enhance ligand binding within protein active pockets. The presence of the bromine atom may therefore strengthen hydrophobic interactions within the kinase binding cavity, potentially contributing to the observed inhibitory effects on MAPK signaling.

Compared with previously reported MAPK inhibitors, QFM-3m is distinguished by its novel dihydrofurobenzofuran scaffold, selective MAPK pathway modulation without broad NF-κB suppression, and inhalational delivery, allowing targeted lung exposure (Notarte et al., 2023; Yang et al., 2022). Integration of transcriptomic profiling, functional validation, and *in silico* docking provides a multi-layered mechanistic understanding that goes beyond conventional cytokine assays. Moreover, unlike meroterpenoids such as rhodomyrtone and rhodomentosones, which broadly inhibit inflammatory signaling, QFM-3m preferentially targets MAPK, potentially reducing the risk of excessive immunosuppression. These features collectively highlight QFM-3m as a promising lead compound for acute lung injury therapy.

## Conclusion

**FIGURE 8:**
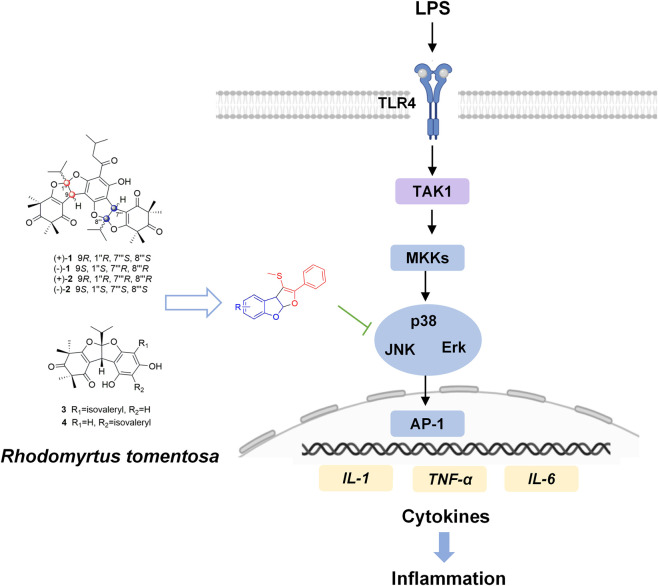
Schematic overview of the discovery and mechanism of action of QFM-3m derived from *Rhodomyrtus tomentosa*. Through rational chemical derivatization of the core scaffold isolated from *Rhodomyrtus tomentosa* extract, a series of 5-substituted dihydrofuro [2,3-b]benzofuran derivatives were designed and screened for anti-inflammatory activity. Among them, the bromine-substituted derivative (designated QFM-3m) was identified as the lead compound. QFM-3m effectively attenuated cytokine release, inflammatory cell infiltration, and lung tissue injury in an LPS-induced murine model of ALI. Mechanistic studies revealed that QFM-3m exerts its anti-inflammatory effects primarily through suppression of MAPK signaling.

In summary, this work translates a traditional medicinal herb through rational chemical derivatization into a novel small-molecule chemotype with *in vivo* efficacy. QFM-3m as a representative member of a new class of dihydrofuro [2,3-b] benzofuran-based compounds with potent anti-inflammatory properties. By inhibiting MAPK signaling, QFM-3m effectively reduces cytokine secretion, inflammatory cell infiltration, and lung injury in LPS-induced ALI (Figure 8). These findings not only underscore the therapeutic potential of QFM-3m but also provide a foundation for the development of structurally novel agents for the treatment of acute lung injury.

## Data Availability

The RNA-seq datasets are available at the NCBI repository, accession number PRJNA139539.
