# Supplementary material for: A dihydrofuro[2,3-b] benzofuran derivative alleviates lipopolysaccharide induced acute lung injury via suppressing MAPK signaling
Source: Front Pharmacol. 2026 Apr 21;17:1763318. doi: 10.3389/fphar.2026.1763318 (PMC13139021; doi:10.3389/fphar.2026.1763318)
Supplement: Supplementary file 2 [file Table1.DOCX]

**Supplemental Table 1. The primer pairs used in the project**

| **Gene name** | **Primer sequence** | **Tm** | **Products(bp)** |
| --- | --- | --- | --- |
| Mice *IL-1α* | 5-TCTGCCATTGACCATCTC - 3 | 58℃ | 182 |
|  | 5-ATCTTCCCGTTGCTTGAC - 3 |  |  |
| Mice *IL-1β* | 5- GTTCCCATTAGACAACTGC- 3 | 60℃ | 199 |
|  | 5-GATTCTTTCCTTTGAGGC - 3 |  |  |
| Mice *IL-6* | 5-TGCCTTCTTGGGACTGAT - 3 | 60℃ | 183 |
|  | 5-TTGCCATTGCACAACTCTTT - 3 |  |  |
| Mice *TNF-α* | 5- CCAGACCCTCACACTCAGAT- 3 | 60℃ | 187 |
|  | 5-GACAAGGTACAACCCATCG - 3 |  |  |
| *β-actin* | 5-CACGATGGAGGGGCCGGACTCATC - 3 | 58℃ | 241 |
|  | 5-TAAAGACCTCTATGCCAACACAGT - 3 |  |  |
